# Genetic and Chemical Effects on Somatic and Germline Aging

**DOI:** 10.1155/2020/4684890

**Published:** 2020-01-04

**Authors:** Myon-Hee Lee, Huai-Rong Luo, Soo Han Bae, Adriana San-Miguel

**Affiliations:** ^1^Department of Internal Medicine (Division of Hematology/Oncology), Brody School of Medicine at East Carolina University, Greenville, NC 27834, USA; ^2^Key Laboratory for Aging and Regenerative Medicine, Department of Pharmacology, School of Pharmacy, Southwest Medical University, Luzhou, Sichuan 646000, China; ^3^Severance Biomedical Science Institute, Yonsei Biomedical Research Institute, Yonsei University College of Medicine, 50 Yonsei-ro, Seodaemun-gu, Seoul 03722, Republic of Korea; ^4^Department of Chemical and Biomolecular Engineering, NC State University, Raleigh, NC 27695, USA

Somatic aging is a complex process characterized by gradual deterioration of physiological function that is observed at the genetic, molecular, and cellular levels. The role of genetic and chemical factors in the aging phenomenon extends to various research fields, including stem cell biology, diabetes, and cancer. Although the fundamental mechanisms behind the aging process are still poorly understood, increasing evidence shows that a progressive and irreversible accumulation of oxidative injury, caused by reactive oxygen species (ROS), impacts negatively on the aging process and contributes to impaired physiological function, increased incidence of age-related disease, and shortened lifespan.

In addition to somatic aging, several studies have shown that reproductive capabilities similarly decline with age (also termed germline aging). Germline tissue is the only tissue designed to support the development of an entire organism, and therefore, it may be the ultimate source of stem cells for tissue replacement in diseased or injured individuals. Specifically, germline aging manifests as diminished germline stem cell (GSC) capacity and reduced germ cell numbers. Several reports have demonstrated that signals from the reproductive system influence somatic aging and *vice versa*. However, mechanisms governing this intricate process remain ill defined.

Therefore, this special issue will focus on the impact of genetic (e.g., miR-22, SIRT-1, AT1R, TGF-*β*1, Smad2/3, daf-12/16, NRF2) and chemical factors (e.g., triclosan, salidroside, folic acid, Lycium barbarum polysaccharide, porphyran, lipidome) on antioxidant defense mechanisms and age-induced somatic and germline aging, using both invertebrate and vertebrate model organisms, which may address fundamental biological questions regarding aging and development.

X. Zhang et al. demonstrated that sleep deprivation induces serious telomere dysfunction and a subsequent senescence-associated secretory phenotype (SASP). In addition, authors showed for the first time that folic acid (also known as vitamin B_9_) supplementation could reverse telomere damage and the secretion of senescence-associated cytokines in both mice and humans.

R. He et al. reported that upregulation of TRPC3 is involved in atrial fibrosis in aging and spontaneously hypertensive rats. Furthermore, pharmacological TRPC3 selected blocker or knockdown of TRPC3 both attenuates Ang II-induced atrial fibroblasts migration, proliferation, and the expression of fibrotic biomarkers. Ca^2+^-permeable TRPC3 mediates atrial fibrosis through the Ca^2+^/AT1R/TGF-*β*1/p-Smad2/3 signaling pathway, suggesting which may be a potential therapeutic target of the pathogenesis of atrial fibrillation during aging and hypertension.

G.-X. Mao et al. studied the effects of salidroside, a compound isolated from Rhodiola rosea L., on cellular senescence. The authors found that salidroside delays cellular senescence in human fibroblasts partly by increasing mitochondrial biogenesis. The authors also show that salidroside induces expression of SIRT1, a necessary step in of mitochondrial biogenesis. Finally, the authors studied the role of the miR-22 miRNA in SIRT1 expression and revealed that increased SIRT1 expression is an in part result of miR-22 inhibition.

Z. Zhang et al. reported that the Lycium barbarum polysaccharides (LBP) extracted from Lycium barbarum show a positive effect on longevity, the abilities to withstand environmental stress, reproduction, and maintenance of muscle integrity mainly through *sir-2.1*, *daf-12*, and *daf-16* using the nematode *C. elegans* as a model system.

M. Chen et al. reported that the proper differentiation of gonad somatic cells is essential for germ cell meiosis. Aberrant development of gonad somatic cells leads to ectopic expression of meiosis-associated genes in germ cells, subsequently causes meiosis arrest before prophase I. In *Wt1^-/flox^*; *Cre-ER™* mice, inactivation of *Wt1* by the injection of tamoxifen at E9.5 blocks the differentiation of Sertoli and granulosa cells and causes most germ cells migrate outside of genital ridge. STRA8, SYCP3, and *γ*H2AX proteins are detected in germ cells of both male and female *Wt1^-/flox^*; *Cre-ER™* gonads, whereas no thread-like SYCP3 signal is observed.

S.-H. Kim et al. reported the antioxidant and antiaging effect of phosphatidylcholine and the underlying mechanism involved in phosphatidylcholine-induced longevity using the nematode *Caenorhabditis elegans* as a model system. Specifically, the authors demonstrated that phosphatidylcholine delays age-related decline of motility by the upregulation of longevity-assurance genes, *hsp-16.2* and *sod-3*.

A. R. Ghanam et al. investigated the age-associated expression of three molecular hallmarks of aging: SA-*β*-gal, P16^INK4a^, and retrotransposable elements (RTEs), in different mouse tissues during chronological aging, and revealed variable expression patterns of these markers in different tissues with aging. P16^INK4a^ shows consistent increases with age in most tissues, while expression of RTEs is variable among different tissues examined. These data suggest that biological changes occurring with physiological aging may be useful in choosing the appropriate timing of therapeutic interventions to slow the aging process or keep more susceptible organs healthier in the aging process.

Q.-L. Wan et al. used a comprehensive high-resolution lipidome to show the relationship between longevity and lipid metabolism using *C. elegans* as a model system. The authors demonstrated that signals from the reproductive tissues may affect animal lifespan at least in part by regulating FOXO/DAF-16-mediated metabolic changes.

M. A. Alfhili and M. H. Lee provide an update on current knowledge regarding the therapeutic and toxic potential of triclosan, a chlorinated phenolic antimicrobial agent and a potential inhibitor of NRF2-mediated oxidative stress response. Specifically, this review emphasizes on the biochemical and molecular alterations, either brought about by or in response to TCS exposure at both the cellular and organismal levels.

